# Octa­kis(di­butyl­ammonium) deca­molybdate(VI)

**DOI:** 10.1107/S2414314624004632

**Published:** 2024-05-31

**Authors:** Papa Aly Gueye, Lamine Yaffa, Dame Seye, Daouda Ndoye, Bocar Traoré, Mamadou Sidibé, Cheikh Abdoul Khadir Diop, Sergiu Shova

**Affiliations:** a Laboratoire de Chimie Minérale et Analytique (LACHIMIA), Département de Chimie, Faculté des Sciences et Techniques, Université Cheikh Anta Diop de Dakar, Senegal; b Département Physique Chimie, UFR Sciences et Technologies, Université Ibra Der Thiam de Thiès, Senegal; c Inorganic Polymers Department, "Petru Poni" Institute of Macramolecular Chemistry, Alea Gr. Ghica Voda 41 A, Iasi 700487, Romania; Vienna University of Technology, Austria

**Keywords:** crystal structure, polyoxidometalate, β-octa­molybdate, hybrid compound

## Abstract

In the title salt, (C_8_H_20_N)_8_[Mo_10_O_34_], the [Mo_10_O_34_]^8−^ polyanion is located about an inversion centre and can be considered as a *β*-type octa­molybdate anion to which two additional MoO_4_ tetra­hedra are linked *via* common corners. The [Mo_10_O_34_]^8−^ polyanions are packed in rows extending parallel to [001] and are connected to the di­butyl­ammonium counter-cations through N—H⋯O hydrogen-bonding inter­actions.

## Abstract 2

(C_8_H_20_N)_8_[Mo_10_O_34_] comprises a centrosymmetric deca­molybdate polyanion linked through N—H⋯O hydrogen bonds to di­butyl­ammonium counter-cations.

## Graphical abstract

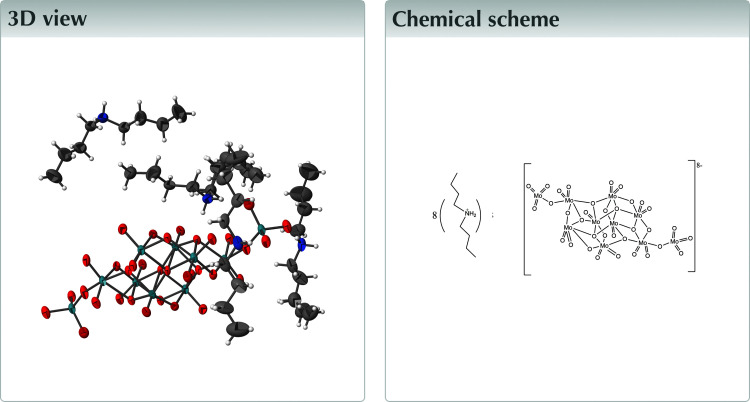

## Structure description

Polyoxometalates (POMs) are obtained by self-assembly of transition-metal oxide units [*M*O_*n*_]^*p*−^ in acidic media (*M* = metal; *n* = 3, 4, 6, ⋯; *p* = 0, 1, 2, 3, ⋯). POMs and their derivatives are an important group of materials that have attracted considerable inter­est in areas such as electrochemistry (Zhang *et al.*, 2021 ▸), materials science (Hao *et al.*, 2007 ▸; Li *et al.*, 2007 ▸), and medicine (Cronin *et al.*, 2002 ▸; Müller *et al.*, 1999 ▸). In recent years, research on organic–inorganic hybrid POMs has experienced significant growth, supported by possible modifications and/or functional­izations of the oxide surface of the POM with preselected organic moieties (Xu *et al.*, 2003 ▸). The structural diversity of the corresponding isopolyoxomolybdates is due to characteristic large polyanionic units and organic ammonium cations, which consolidate the crystal structures through non-covalent supra­molecular inter­actions. In this regard, several octa­molybdate polyanions [Mo_8_O_26_]^4–^, charge-balanced by organic counter-ions, have been synthesized and structurally characterized (Allis *et al.*, 2004 ▸; Harchani & Haddad, 2015 ▸). For the current study, we used diiso­butyl­ammonium as a counter-cation and obtained the hybrid organic–inorganic deca­molybdate (C_8_H_20_N)_8_[Mo_10_O_34_].

**Figure 1:**
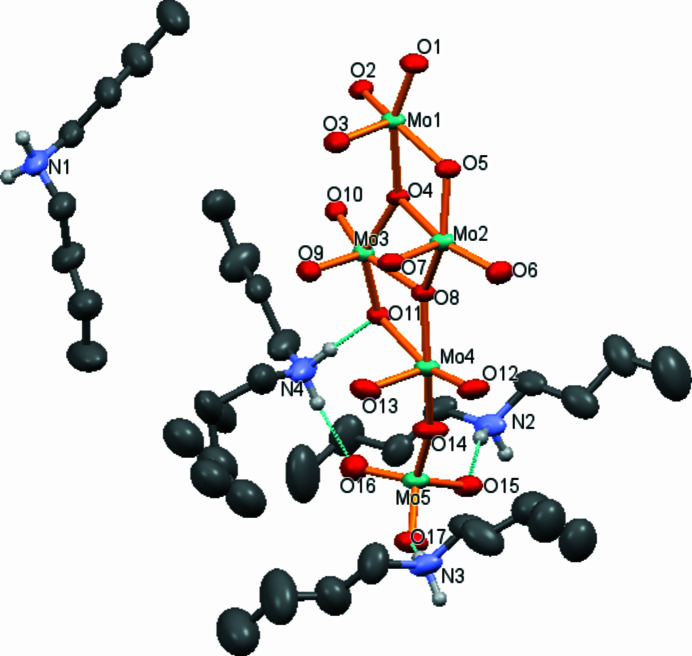
The asymmetric unit of the title compound. Displacement ellipsoids are drawn at the 50% probability level. Dotted lines indicate N—H⋯O hydrogen-bonding inter­actions. The C-bound H atoms are omitted for clarity.

**Figure 2:**
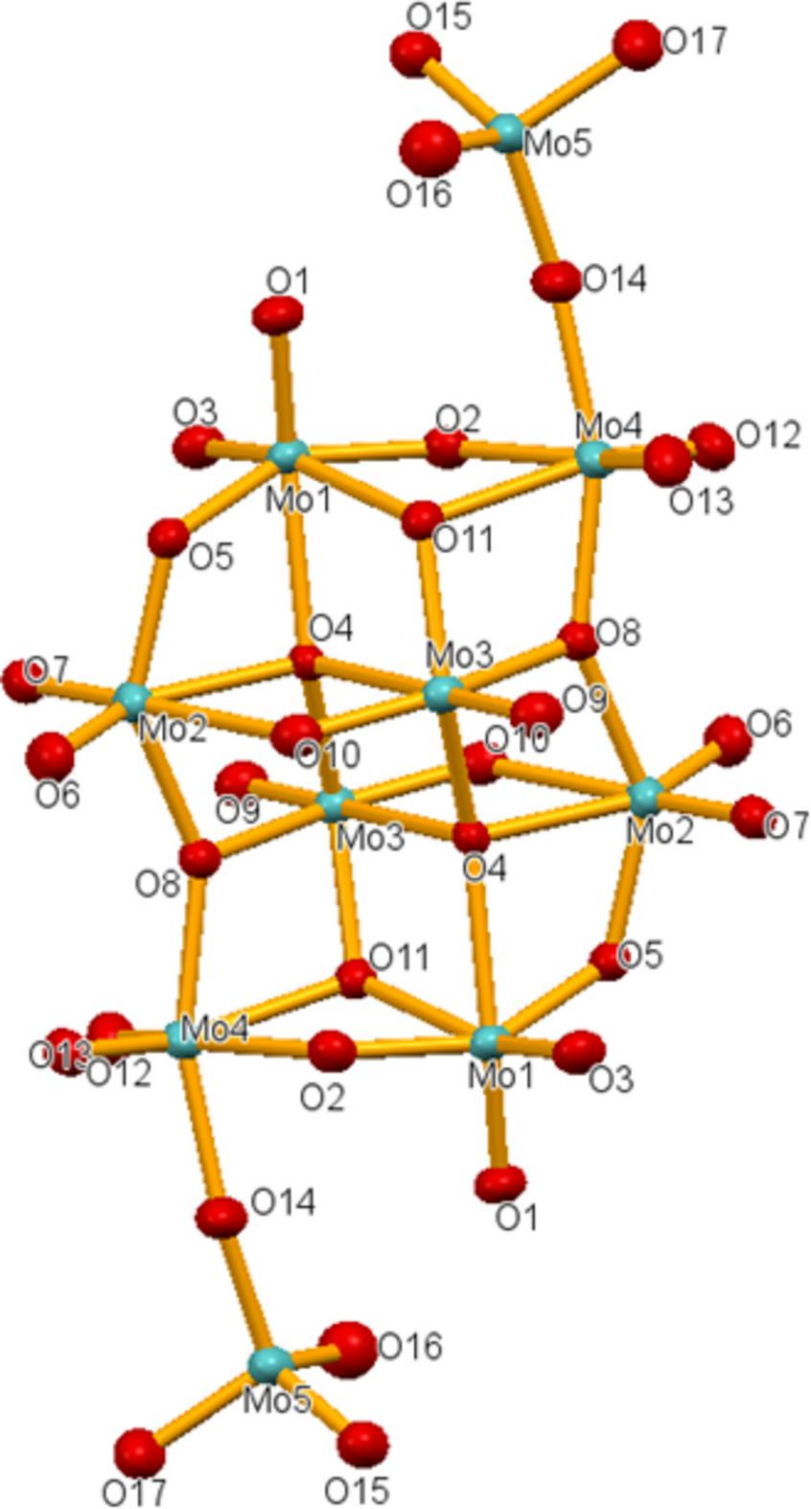
The centrosymmetric [Mo_10_O_34_]^8–^ polyanion in the title compound. Displacement ellipsoids are drawn at the 50% probability level.

The asymmetric unit of (C_8_H_20_N)_8_[Mo_10_O_34_] is shown in Fig. 1 ▸. The [Mo_10_O_34_]^8–^ anion is located about an inversion centre and is displayed in Fig. 2 ▸. Such kind of deca­molybdate anion is known from other ammonium salts and has been reported for the first time for (NH_4_)_8_[Mo_10_O_34_] (Fuchs *et al.*, 1975 ▸). The [Mo_10_O_34_]^8–^ anion can be considered as a *β*-type octa­molydate to which two additional MoO_4_ tetra­hedra are added *via* vertex-sharing. Two types of *β*-octa­molybdate anions can be distinguished, type *A* with the general formula [Mo_8_O_26_]^4–^ and type *B* with the general formula [H_*x*_Mo_8_O_28_]^(8–*x*)^ (Pavani *et al.*, 2007 ▸). Thus, the [Mo_10_O_34_]^8–^ anion of the title compound can be considered as of the *β*-octa­molybdate *B* type (Du *et al.* 2011 ▸; Isobe *et al.* 1978 ▸).

The [Mo_10_O_34_]^8–^ polyanion is made up of eight MoO_6_ octa­hedra linked to each other by edge and/or vertex sharing, building up an octa­molybdate anion. Similar POMs with an Mo_8_ core linked to the ends by Mo_*x*_O_*y*_ groups are found in the crystal structures of [NH_3_(CH_2_)_2_NH_2_(CH_2_)_2_NH_3_]_2_[Mo_9_O_30_] and [NH_3_(CH_2_)_2_NH_2_(CH_2_)_3_NH_3_]_2_[Mo_10_O_33_] (Chakrabarti & Natarajan, 2002 ▸). In (C_8_H_20_N)_8_[Mo_10_O_34_], the *β*-octa­molybdate polyanion is linked with two additional MoO_4_ tetra­hedra, which can be expressed by the formula [(MoO_3_)_2_ *β*-Mo_8_O_28_]^8–^. Bond-valence calculations show that the five crystallographically unique Mo atoms are in the +VI oxidation state. According to the role of the oxygen ligands (terminal or bridging) in the *β*-octa­molybdate moiety, the corresponding Mo—O bond lengths for Mo1–Mo4 range from 1.703 (3) to 2.451 (3) Å and the O—Mo—O bond angles from 71.01 (12) to 179.57 (14)°. These values are in the range expected for octa­hedrally coordinated Mo^VI^ atoms and in agreement with those in the previously reported octa­molybdate structure. (Pavani & Ramanan, 2005 ▸; Wu *et al.*, 2002 ▸). The Mo5 site is tetra­hedrally surrounded by three terminal oxygen atoms (O15, O16, O17) with bond lengths between 1.738 (4) and 1.767 (4) Å and a bridging oxygen atom O14 to the *β*-octa­molybdate anion with 1.804 (4) Ā. The angles of the tetra­hedron range from 107.5 (2) to 112.0 (2)°.

**Figure 3:**
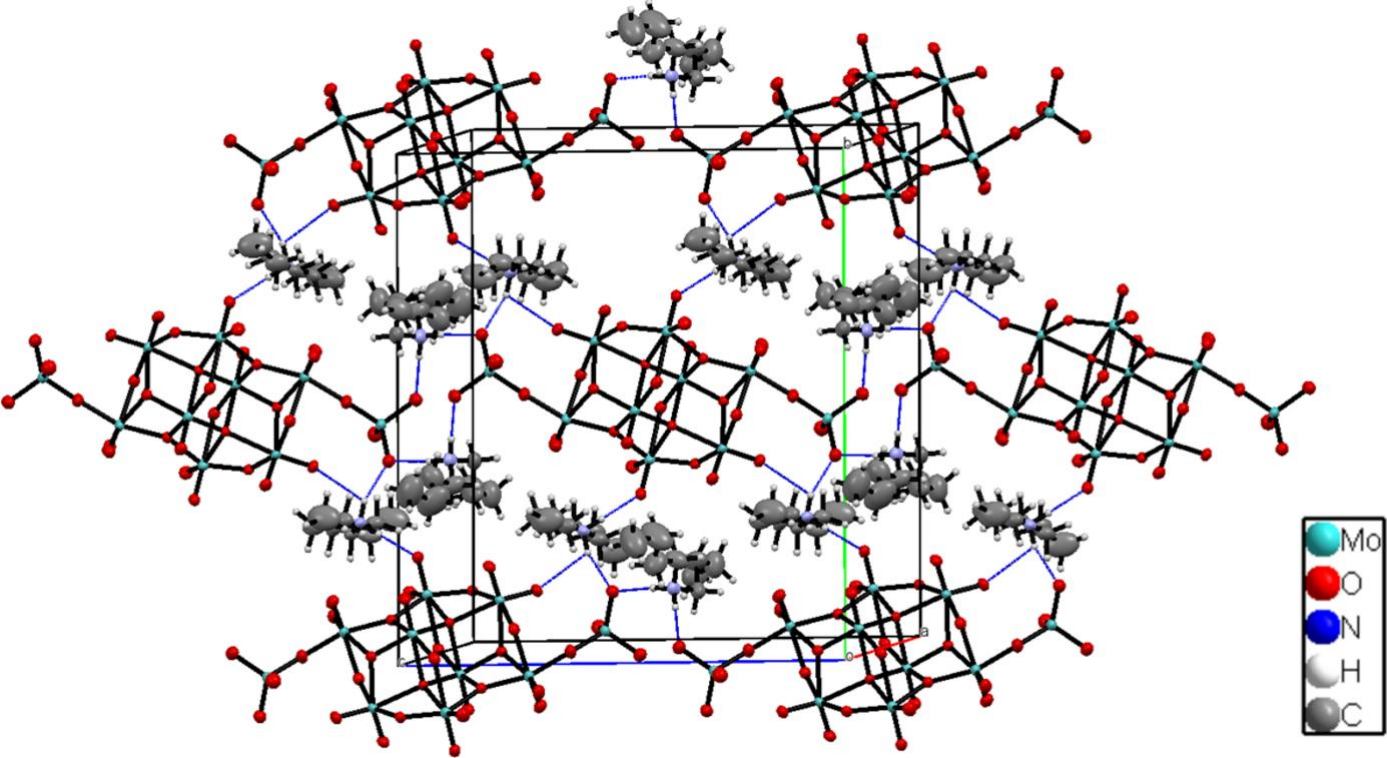
The unit-cell packing viewed down [001] with hydrogen bonds indicated by blue dashed lines.

In the crystal, the [Mo_10_O_34_]^8–^ polyanions are stacked into rows parallel to [001] and surrounded by di­butyl­ammonium counter-cations. Next to Coulombic inter­actions, cations and anions are linked through rather strong N—H**⋯**O hydrogen bonds between the ammonium cations and the terminal oxygen atoms of the MoO_4_ tetra­hedra (O15⋯H3*A*—N3 and O17⋯H3*B*—N3; Table 1 ▸, Fig. 3 ▸). The other ammonium groups are involved in hydrogen-bonding inter­actions with the terminal O atoms of the *β*-octa­molybdate moiety (Table 1 ▸).

**Figure 4:**
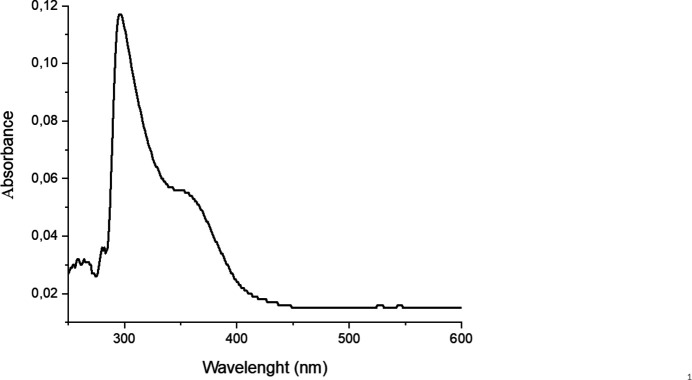
UV/Vis spectrum of the title compound.

The UV-vis absorption spectrum of the title compound was recorded in the range 250–700 nm in aqueous solution (0.1 *N*) and is shown in Fig. 4 ▸. It shows two absorption bands at 297 nm and 353 nm. The strongest band at 297 nm is attributed to a charge-transfer transition of the type O_t_ —Mo and the shoulder peak at 353 nm to a charge-transfer transition of the type Mo—O—Mo (Gong *et al.*, 2006 ▸; Zhang *et al.*, 1997 ▸)

## Synthesis and crystallization

Ammonium hepta­molybdate, (NH_4_)_6_[Mo_7_O_24_]·4H_2_O (4.943 g), and di­butyl­amine, C_8_H_19_N (1.559 g), were dissolved in 40 ml of hot water. The mixture was heated for 2 h at 473 K under reflux and then filtered. The filtrate was kept for three months at ambient conditions, affording colourless crystals in about 8% yield (based on Mo).

## Refinement

Crystal data, data collection and structure refinement details are summarized in Table 2 ▸.

## Supplementary Material

Crystal structure: contains datablock(s) I. DOI: 10.1107/S2414314624004632/wm4210sup1.cif

Structure factors: contains datablock(s) I. DOI: 10.1107/S2414314624004632/wm4210Isup4.hkl

CCDC reference: 2332866

Additional supporting information:  crystallographic information; 3D view; checkCIF report

## Figures and Tables

**Table 1:** Hydrogen-bond geometry (Å, °)

*D*—H⋯*A*	*D*—H	H⋯*A*	*D*⋯*A*	*D*—H⋯*A*
N1—H1*A*⋯O2^i^	0.91	1.72	2.627 (6)	172
N1—H1*B*⋯O10^i^	0.91	1.97	2.755 (6)	143
N2—H2*C*⋯O15	0.91	1.91	2.778 (6)	160
N2—H2*D*⋯O7^ii^	0.91	1.90	2.812 (5)	176
N3—H3*A*⋯O15^iii^	0.91	1.87	2.770 (6)	172
N3—H3*B*⋯O17	0.91	1.88	2.786 (6)	176
N4—H4*A*⋯O11	0.91	1.80	2.695 (6)	166
N4—H4*B*⋯O16	0.91	1.86	2.762 (7)	172

Symmetry codes: (i) 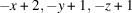 ; (ii) 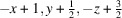 ; (iii) 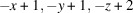 .

**Table 2:** Experimental details

Crystal data	
Chemical formula	(C_8_H_20_N)_8_[Mo_10_O_34_]
*M*_r_	2545.39
Crystal system, space group	Monoclinic, *P*2_1_/*c*
Temperature (K)	130
*a*, *b*, *c* (Å)	14.21628 (18), 20.7477 (2), 18.2210 (2)
β (°)	110.0785 (15)
*V* (Å^3^)	5047.74 (12)
*Z*	2
Radiation type	Cu *K*α
μ (mm^−1^)	10.44
Crystal size (mm)	0.15 × 0.03 × 0.02

Data collection	
Diffractometer	XtaLAB Synergy, Dualflex, HyPix
Absorption correction	Multi-scan (*CrysAlis PRO*; Rigaku OD, 2023 ▸)
*T*_min_, *T*_max_	0.500, 1.000
No. of measured, independent and observed [*I* > 2σ(*I*)] reflections	42024, 9853, 8753
*R*_int_	0.043
(sin θ/λ)_max_ (Å^−1^)	0.625

Refinement	
*R*[*F* ^2^ > 2σ(*F* ^2^)], *wR*(*F* ^2^), *S*	0.045, 0.119, 1.02
No. of reflections	9853
No. of parameters	541
No. of restraints	18
H-atom treatment	H-atom parameters constrained
Δρ_max_, Δρ_min_ (e Å^−3^)	1.65, −1.09

Computer programs: *CrysAlis PRO* (Rigaku OD, 2023 ▸), *SHELXT* (Sheldrick, 2015*a* ▸), *SHELXL* (Sheldrick, 2015*b* ▸) and *OLEX2* (Dolomanov *et al.*, 2009 ▸).

## full crystallographic data

### Crystal data

**Table:**

(C_8_H_20_N)_8_[Mo_10_O_34_]	*F*(000) = 2584
*M**_r_* = 2545.39	*D*_x_ = 1.675 Mg m^−^^3^
Monoclinic, *P*2_1_/*c*	Cu *K*α radiation, λ = 1.54184 Å
*a* = 14.21628 (18) Å	Cell parameters from 25536 reflections
*b* = 20.7477 (2) Å	θ = 3.3–76.7°
*c* = 18.2210 (2) Å	µ = 10.44 mm^−^^1^
β = 110.0785 (15)°	*T* = 130 K
*V* = 5047.74 (12) Å^3^	Needle, clear colourless
*Z* = 2	0.15 × 0.03 × 0.02 mm

### Data collection

**Table:**

XtaLAB Synergy, Dualflex, HyPix diffractometer	9853 independent reflections
Radiation source: micro-focus sealed X-ray tube, PhotonJet (Cu) X-ray Source	8753 reflections with *I* > 2σ(*I*)
Mirror monochromator	*R*_int_ = 0.043
Detector resolution: 10.0000 pixels mm^-1^	θ_max_ = 74.5°, θ_min_ = 3.3°
ω scans	*h* = −17→14
Absorption correction: multi-scan (CrysAlisPro; Rigaku OD, 2023)	*k* = −24→24
*T*_min_ = 0.500, *T*_max_ = 1.000	*l* = −22→22
42024 measured reflections	

### Refinement

**Table:**

Refinement on *F*^2^	Primary atom site location: dual
Least-squares matrix: full	Hydrogen site location: inferred from neighbouring sites
*R*[*F*^2^ > 2σ(*F*^2^)] = 0.045	H-atom parameters constrained
*wR*(*F*^2^) = 0.119	*w* = 1/[σ^2^(*F*_o_^2^) + (0.0685*P*)^2^ + 16.9839*P*] where *P* = (*F*_o_^2^ + 2*F*_c_^2^)/3
*S* = 1.02	(Δ/σ)_max_ = 0.001
9853 reflections	Δρ_max_ = 1.65 e Å^−^^3^
541 parameters	Δρ_min_ = −1.09 e Å^−^^3^
18 restraints	

### Special details

**Table:**

Geometry. All esds (except the esd in the dihedral angle between two l.s. planes) are estimated using the full covariance matrix. The cell esds are taken into account individually in the estimation of esds in distances, angles and torsion angles; correlations between esds in cell parameters are only used when they are defined by crystal symmetry. An approximate (isotropic) treatment of cell esds is used for estimating esds involving l.s. planes.

### Fractional atomic coordinates and isotropic or equivalent isotropic displacement parameters (Å^2^)

**Table:**

	*x*	*y*	*z*	*U*_iso_*/*U*_eq_	Occ. (<1)
Mo1	0.46738 (3)	0.39489 (2)	0.35188 (2)	0.02715 (10)	
Mo2	0.39496 (3)	0.38023 (2)	0.50285 (2)	0.02920 (10)	
Mo3	0.60253 (3)	0.46487 (2)	0.57062 (2)	0.02504 (10)	
Mo4	0.50325 (3)	0.45478 (2)	0.70777 (2)	0.02984 (10)	
Mo5	0.60971 (3)	0.54579 (2)	0.89668 (2)	0.03279 (11)	
O1	0.3980 (3)	0.37372 (16)	0.25734 (19)	0.0367 (8)	
O2	0.5564 (3)	0.45633 (15)	0.33548 (19)	0.0323 (7)	
O3	0.5479 (3)	0.33261 (16)	0.3883 (2)	0.0358 (7)	
O4	0.5043 (2)	0.43977 (14)	0.47119 (17)	0.0248 (6)	
O5	0.3602 (3)	0.37118 (15)	0.39495 (18)	0.0298 (7)	
O6	0.2865 (3)	0.37049 (17)	0.5231 (2)	0.0386 (8)	
O7	0.4603 (3)	0.30910 (16)	0.53384 (19)	0.0343 (7)	
O8	0.4764 (2)	0.43107 (15)	0.59639 (18)	0.0284 (6)	
O9	0.6765 (3)	0.39936 (16)	0.60011 (19)	0.0345 (7)	
O10	0.6746 (2)	0.51676 (16)	0.53399 (19)	0.0305 (7)	
O11	0.6152 (2)	0.50842 (14)	0.66534 (18)	0.0284 (6)	
O12	0.3977 (3)	0.41825 (18)	0.7121 (2)	0.0425 (9)	
O13	0.5989 (3)	0.39987 (17)	0.7488 (2)	0.0419 (8)	
O14	0.5422 (4)	0.50320 (18)	0.8079 (2)	0.0483 (10)	
O15	0.5518 (3)	0.61967 (17)	0.9041 (2)	0.0406 (8)	
O16	0.7336 (3)	0.5584 (2)	0.9045 (2)	0.0486 (9)	
O17	0.6081 (3)	0.49797 (18)	0.9747 (2)	0.0473 (9)	
N1	1.2542 (4)	0.5261 (3)	0.5803 (3)	0.0528 (13)	
H1A	1.317838	0.533197	0.613310	0.063*	
H1B	1.257981	0.498154	0.542852	0.063*	
C1	1.0075 (8)	0.6326 (6)	0.3483 (6)	0.103 (4)	
H1C	1.003411	0.670808	0.315617	0.155*	
H1D	0.944901	0.627889	0.358998	0.155*	
H1E	1.018427	0.594290	0.320836	0.155*	
C2	1.0942 (7)	0.6403 (5)	0.4248 (5)	0.087 (3)	
H2A	1.152069	0.659291	0.414106	0.104*	
H2B	1.074403	0.670627	0.458930	0.104*	
C3	1.1249 (5)	0.5784 (4)	0.4665 (4)	0.068 (2)	
H3C	1.143849	0.547696	0.432336	0.081*	
H3D	1.067847	0.559794	0.478613	0.081*	
C4	1.2132 (5)	0.5878 (4)	0.5420 (4)	0.0581 (16)	
H4C	1.266756	0.611691	0.530394	0.070*	
H4D	1.191445	0.614235	0.578467	0.070*	
C5	1.1975 (5)	0.4946 (4)	0.6249 (4)	0.0613 (17)	
H5A	1.217167	0.448646	0.633571	0.074*	
H5B	1.124920	0.496506	0.594592	0.074*	
C6	1.2188 (7)	0.5285 (5)	0.7041 (5)	0.078 (2)	
H6A	1.292066	0.533544	0.729856	0.093*	
H6B	1.188595	0.572041	0.695217	0.093*	
C7	1.1781 (8)	0.4919 (6)	0.7566 (6)	0.097 (3)	
H7A	1.204890	0.447427	0.763302	0.116*	
H7B	1.104202	0.489454	0.733008	0.116*	
C8	1.2075 (11)	0.5256 (7)	0.8371 (6)	0.132 (5)	
H8A	1.164148	0.563035	0.833396	0.197*	
H8B	1.277455	0.539697	0.853090	0.197*	
H8C	1.199623	0.495291	0.875880	0.197*	
N2	0.5610 (4)	0.7400 (2)	0.8400 (3)	0.0435 (11)	
H2C	0.558726	0.697106	0.849875	0.052*	
H2D	0.551162	0.761770	0.880137	0.052*	
C9	0.9281 (8)	0.7162 (6)	0.9733 (10)	0.136 (5)	
H9A	0.925536	0.730830	1.023715	0.204*	
H9B	0.993739	0.726632	0.969919	0.204*	
H9C	0.917704	0.669441	0.968751	0.204*	
C10	0.8498 (7)	0.7484 (4)	0.9101 (6)	0.083 (3)	
H10A	0.856978	0.736603	0.859627	0.099*	
H10B	0.858736	0.795591	0.916640	0.099*	
C11	0.7436 (5)	0.7304 (3)	0.9077 (4)	0.0569 (16)	
H11A	0.737917	0.682872	0.908117	0.068*	
H11B	0.733897	0.747021	0.955539	0.068*	
C12	0.6631 (5)	0.7559 (3)	0.8388 (4)	0.0496 (14)	
H12A	0.670511	0.737780	0.790857	0.059*	
H12B	0.669932	0.803313	0.837023	0.059*	
C13	0.4791 (5)	0.7553 (3)	0.7679 (3)	0.0521 (15)	
H13A	0.487501	0.730037	0.724489	0.063*	
H13B	0.482772	0.801585	0.755759	0.063*	
C14	0.3794 (6)	0.7416 (4)	0.7727 (5)	0.071 (2)	
H14A	0.369298	0.768421	0.814244	0.085*	
H14B	0.376330	0.695772	0.786885	0.085*	
C15	0.2959 (6)	0.7553 (4)	0.6953 (6)	0.085 (3)	
H15A	0.301443	0.800569	0.680112	0.102*	
H15B	0.305073	0.727183	0.654378	0.102*	
C16	0.1939 (9)	0.7446 (7)	0.6986 (9)	0.138 (5)	
H16A	0.187367	0.767691	0.743506	0.207*	
H16B	0.183356	0.698427	0.703895	0.207*	
H16C	0.143702	0.760723	0.650403	0.207*	
N3	0.5165 (4)	0.3786 (2)	0.9706 (3)	0.0484 (12)	
H3A	0.498144	0.376434	1.013761	0.058*	
H3B	0.548926	0.416775	0.972730	0.058*	
C18	0.2616 (9)	0.3213 (6)	0.8363 (6)	0.105 (3)	
H18A	0.261341	0.352706	0.795295	0.126*	0.333 (15)
H18B	0.244014	0.278560	0.811063	0.126*	0.333 (15)
H18C	0.227094	0.359599	0.847226	0.126*	0.667 (15)
H18D	0.267273	0.327310	0.784033	0.126*	0.667 (15)
C19	0.3625 (8)	0.3181 (4)	0.8945 (6)	0.091 (3)	
H19A	0.398549	0.281599	0.881310	0.110*	
H19B	0.356831	0.309039	0.946131	0.110*	
C20	0.4248 (7)	0.3793 (4)	0.9009 (4)	0.0668 (19)	
H20A	0.443553	0.383491	0.853603	0.080*	
H20B	0.383793	0.417232	0.903352	0.080*	
C21	0.5880 (6)	0.3257 (4)	0.9748 (6)	0.080 (2)	
H21A	0.558598	0.284238	0.983043	0.096*	
H21B	0.600695	0.322988	0.924752	0.096*	
C22	0.6830 (8)	0.3368 (6)	1.0391 (7)	0.102 (3)	
H22A	0.668767	0.339739	1.088525	0.123*	
H22B	0.710307	0.378925	1.030655	0.123*	
C23	0.7605 (11)	0.2871 (8)	1.0484 (10)	0.156 (6)	
H23A	0.775827	0.284082	0.999411	0.187*	
H23B	0.734182	0.244800	1.057416	0.187*	
C24	0.8592 (12)	0.3022 (9)	1.1178 (9)	0.163 (7)	
H24A	0.891581	0.340187	1.105114	0.245*	
H24B	0.904591	0.265168	1.126657	0.245*	
H24C	0.843257	0.310613	1.165148	0.245*	
C17X	0.189 (2)	0.3390 (16)	0.868 (2)	0.106 (5)	0.333 (15)
H17A	0.124953	0.344533	0.825605	0.159*	0.333 (15)
H17B	0.208992	0.379747	0.896216	0.159*	0.333 (15)
H17C	0.183507	0.305427	0.903659	0.159*	0.333 (15)
C17	0.2031 (13)	0.2674 (8)	0.8340 (12)	0.106 (5)	0.667 (15)
H17D	0.234106	0.229559	0.819533	0.159*	0.667 (15)
H17E	0.136177	0.273997	0.795289	0.159*	0.667 (15)
H17F	0.197592	0.260561	0.885594	0.159*	0.667 (15)
N4	0.7926 (4)	0.5318 (3)	0.7782 (3)	0.0639 (17)	
H4A	0.733073	0.530722	0.737974	0.077*	
H4B	0.779178	0.542145	0.822093	0.077*	
C25	0.9612 (7)	0.6165 (5)	0.5961 (6)	0.088 (3)	
H25A	0.897792	0.615423	0.552036	0.132*	
H25B	0.997253	0.575933	0.598168	0.132*	
H25C	1.002122	0.652488	0.589347	0.132*	
C26	0.9400 (8)	0.6254 (5)	0.6717 (7)	0.097 (3)	
H26A	1.004535	0.628966	0.715395	0.116*	
H26B	0.903308	0.666367	0.668837	0.116*	
C27	0.8807 (5)	0.5722 (4)	0.6890 (4)	0.0587 (17)	
H27A	0.919594	0.531675	0.695964	0.070*	
H27B	0.818304	0.566349	0.643671	0.070*	
C28	0.8530 (6)	0.5842 (5)	0.7627 (5)	0.076 (2)	
H28A	0.915332	0.588959	0.808361	0.091*	
H28B	0.815198	0.625050	0.756318	0.091*	
C29	0.8358 (6)	0.4650 (5)	0.7886 (5)	0.084 (3)	
H29C	0.851243	0.451059	0.742069	0.100*	0.354 (12)
H29D	0.789472	0.433582	0.798999	0.100*	0.354 (12)
H29A	0.787341	0.437231	0.801932	0.100*	0.646 (12)
H29B	0.834884	0.450959	0.736475	0.100*	0.646 (12)
C30	0.9371 (9)	0.4479 (14)	0.8450 (7)	0.116 (9)	0.646 (12)
H30A	0.951345	0.402023	0.838008	0.140*	0.646 (12)
H30B	0.988524	0.474333	0.833678	0.140*	0.646 (12)
C31	0.9435 (14)	0.4588 (10)	0.9272 (9)	0.118 (4)	0.646 (12)
H31A	0.928863	0.504727	0.933306	0.142*	0.646 (12)
H31B	1.013140	0.450351	0.961847	0.142*	0.646 (12)
C32	0.8742 (14)	0.4181 (10)	0.9538 (11)	0.118 (4)	0.646 (12)
H32A	0.811153	0.411778	0.910258	0.178*	0.646 (12)
H32B	0.860589	0.439740	0.996901	0.178*	0.646 (12)
H32C	0.905380	0.376152	0.971561	0.178*	0.646 (12)
C30X	0.930 (2)	0.4727 (16)	0.8584 (18)	0.116 (9)	0.354 (12)
H30C	0.914077	0.496308	0.900096	0.140*	0.354 (12)
H30D	0.979275	0.498075	0.843703	0.140*	0.354 (12)
C31X	0.973 (2)	0.4078 (16)	0.888 (2)	0.118 (4)	0.354 (12)
H31C	1.040851	0.413603	0.926996	0.142*	0.354 (12)
H31D	0.978545	0.382011	0.844473	0.142*	0.354 (12)
C32X	0.910 (2)	0.3715 (18)	0.926 (2)	0.118 (4)	0.354 (12)
H32D	0.846812	0.358446	0.886039	0.178*	0.354 (12)
H32E	0.895563	0.399259	0.964503	0.178*	0.354 (12)
H32F	0.946241	0.333117	0.952337	0.178*	0.354 (12)

### Atomic displacement parameters (Å^2^)

**Table:**

	*U*^11^	*U*^22^	*U*^33^	*U*^12^	*U*^13^	*U*^23^
Mo1	0.0428 (2)	0.01922 (17)	0.02010 (17)	0.00043 (13)	0.01172 (15)	−0.00267 (12)
Mo2	0.0424 (2)	0.02309 (19)	0.02335 (18)	−0.00671 (14)	0.01292 (15)	−0.00140 (13)
Mo3	0.03428 (19)	0.01990 (17)	0.02015 (17)	0.00191 (13)	0.00831 (14)	−0.00127 (12)
Mo4	0.0462 (2)	0.02416 (19)	0.01977 (18)	−0.00317 (14)	0.01209 (15)	−0.00028 (13)
Mo5	0.0532 (2)	0.02420 (19)	0.02242 (19)	−0.00053 (15)	0.01481 (17)	−0.00126 (13)
O1	0.056 (2)	0.0296 (17)	0.0240 (16)	−0.0013 (15)	0.0126 (15)	−0.0059 (13)
O2	0.0432 (19)	0.0299 (17)	0.0275 (16)	0.0011 (13)	0.0169 (14)	−0.0018 (13)
O3	0.052 (2)	0.0268 (17)	0.0303 (17)	0.0063 (14)	0.0166 (15)	−0.0031 (13)
O4	0.0383 (16)	0.0193 (14)	0.0198 (14)	−0.0021 (12)	0.0137 (12)	0.0006 (11)
O5	0.0451 (18)	0.0216 (15)	0.0237 (15)	−0.0047 (13)	0.0130 (14)	−0.0026 (12)
O6	0.047 (2)	0.0343 (19)	0.0380 (19)	−0.0090 (15)	0.0195 (16)	−0.0030 (15)
O7	0.052 (2)	0.0274 (17)	0.0259 (16)	−0.0035 (14)	0.0164 (15)	0.0004 (13)
O8	0.0424 (18)	0.0222 (15)	0.0220 (15)	−0.0007 (13)	0.0130 (13)	0.0001 (12)
O9	0.0438 (19)	0.0296 (17)	0.0290 (17)	0.0063 (14)	0.0110 (14)	−0.0011 (13)
O10	0.0340 (17)	0.0302 (17)	0.0277 (16)	0.0014 (13)	0.0111 (13)	−0.0039 (13)
O11	0.0402 (17)	0.0216 (15)	0.0224 (15)	−0.0015 (12)	0.0097 (13)	−0.0010 (12)
O12	0.065 (2)	0.037 (2)	0.0317 (18)	−0.0111 (17)	0.0241 (17)	−0.0015 (15)
O13	0.061 (2)	0.0318 (18)	0.0295 (18)	−0.0034 (16)	0.0103 (16)	0.0049 (14)
O14	0.086 (3)	0.0331 (19)	0.0272 (18)	−0.0084 (19)	0.0214 (19)	−0.0041 (15)
O15	0.062 (2)	0.0308 (18)	0.0322 (18)	0.0013 (16)	0.0202 (17)	−0.0009 (14)
O16	0.057 (2)	0.044 (2)	0.045 (2)	0.0032 (18)	0.0185 (19)	−0.0007 (18)
O17	0.076 (3)	0.0330 (19)	0.0311 (19)	−0.0027 (18)	0.0162 (18)	0.0021 (15)
N1	0.042 (3)	0.078 (4)	0.041 (3)	0.005 (2)	0.017 (2)	−0.016 (3)
C1	0.089 (7)	0.137 (9)	0.067 (6)	0.042 (6)	0.005 (5)	−0.010 (6)
C2	0.082 (6)	0.102 (7)	0.070 (5)	0.033 (5)	0.018 (4)	−0.002 (5)
C3	0.054 (4)	0.088 (6)	0.060 (4)	0.018 (4)	0.017 (3)	−0.008 (4)
C4	0.046 (3)	0.082 (5)	0.048 (3)	0.009 (3)	0.018 (3)	−0.009 (3)
C5	0.052 (4)	0.080 (5)	0.054 (4)	0.003 (3)	0.021 (3)	−0.007 (3)
C6	0.091 (6)	0.087 (6)	0.068 (5)	0.018 (5)	0.043 (5)	−0.006 (4)
C7	0.088 (6)	0.138 (9)	0.069 (6)	0.022 (6)	0.034 (5)	0.010 (6)
C8	0.148 (11)	0.186 (13)	0.058 (6)	0.072 (10)	0.031 (6)	−0.005 (7)
N2	0.075 (3)	0.024 (2)	0.035 (2)	0.003 (2)	0.022 (2)	−0.0035 (17)
C9	0.067 (6)	0.115 (10)	0.221 (17)	−0.008 (6)	0.044 (8)	0.018 (10)
C10	0.080 (5)	0.057 (5)	0.125 (8)	−0.010 (4)	0.051 (6)	0.006 (5)
C11	0.071 (4)	0.034 (3)	0.075 (5)	−0.003 (3)	0.037 (4)	0.000 (3)
C12	0.082 (4)	0.028 (3)	0.051 (3)	−0.007 (3)	0.039 (3)	−0.008 (2)
C13	0.082 (4)	0.033 (3)	0.039 (3)	0.007 (3)	0.018 (3)	−0.007 (2)
C14	0.089 (5)	0.044 (4)	0.064 (5)	−0.003 (3)	0.005 (4)	−0.002 (3)
C15	0.070 (5)	0.064 (5)	0.095 (7)	0.000 (4)	−0.005 (4)	−0.012 (4)
C16	0.086 (8)	0.120 (10)	0.179 (15)	−0.008 (7)	0.010 (8)	0.010 (10)
N3	0.076 (3)	0.040 (3)	0.042 (3)	−0.004 (2)	0.035 (3)	−0.001 (2)
C18	0.103 (8)	0.138 (10)	0.080 (7)	0.005 (7)	0.038 (6)	0.004 (6)
C19	0.108 (7)	0.066 (5)	0.074 (6)	−0.014 (5)	−0.002 (5)	0.000 (4)
C20	0.103 (6)	0.054 (4)	0.042 (4)	0.008 (4)	0.023 (4)	0.000 (3)
C21	0.071 (5)	0.064 (5)	0.109 (7)	0.000 (4)	0.035 (5)	−0.018 (5)
C22	0.090 (7)	0.112 (8)	0.100 (8)	0.013 (6)	0.026 (6)	0.019 (6)
C23	0.120 (10)	0.151 (13)	0.176 (15)	0.045 (9)	0.025 (10)	−0.044 (11)
C24	0.143 (13)	0.201 (18)	0.128 (12)	0.051 (12)	0.023 (10)	−0.022 (12)
C17X	0.094 (10)	0.100 (10)	0.135 (14)	−0.002 (8)	0.053 (9)	0.015 (9)
C17	0.094 (10)	0.100 (10)	0.135 (14)	−0.002 (8)	0.053 (9)	0.015 (9)
N4	0.040 (3)	0.114 (5)	0.038 (3)	−0.007 (3)	0.013 (2)	−0.019 (3)
C25	0.063 (5)	0.129 (8)	0.076 (6)	−0.003 (5)	0.029 (4)	0.022 (5)
C26	0.090 (6)	0.107 (8)	0.106 (8)	−0.035 (6)	0.051 (6)	−0.028 (6)
C27	0.039 (3)	0.085 (5)	0.047 (3)	0.001 (3)	0.009 (3)	−0.007 (3)
C28	0.055 (4)	0.116 (7)	0.057 (4)	−0.021 (4)	0.019 (3)	−0.034 (4)
C29	0.058 (4)	0.131 (8)	0.065 (5)	0.012 (5)	0.025 (4)	0.019 (5)
C30	0.058 (6)	0.20 (2)	0.088 (10)	0.030 (9)	0.021 (6)	0.058 (12)
C31	0.110 (9)	0.138 (12)	0.098 (9)	0.018 (7)	0.023 (6)	0.028 (8)
C32	0.110 (9)	0.138 (12)	0.098 (9)	0.018 (7)	0.023 (6)	0.028 (8)
C30X	0.058 (6)	0.20 (2)	0.088 (10)	0.030 (9)	0.021 (6)	0.058 (12)
C31X	0.110 (9)	0.138 (12)	0.098 (9)	0.018 (7)	0.023 (6)	0.028 (8)
C32X	0.110 (9)	0.138 (12)	0.098 (9)	0.018 (7)	0.023 (6)	0.028 (8)

### Geometric parameters (Å, º)

**Table:**

Mo1—O1	1.723 (3)	C15—H15B	0.9900
Mo1—O2	1.891 (3)	C15—C16	1.489 (16)
Mo1—O3	1.703 (3)	C16—H16A	0.9800
Mo1—O4	2.255 (3)	C16—H16B	0.9800
Mo1—O5	2.001 (3)	C16—H16C	0.9800
Mo1—O11^i^	2.291 (3)	N3—H3A	0.9100
Mo2—O4	2.211 (3)	N3—H3B	0.9100
Mo2—O5	1.865 (3)	N3—C20	1.475 (10)
Mo2—O6	1.717 (4)	N3—C21	1.481 (9)
Mo2—O7	1.732 (3)	C18—H18A	0.9900
Mo2—O8	2.001 (3)	C18—H18B	0.9900
Mo2—O10^i^	2.354 (3)	C18—H18C	0.9900
Mo3—O4	1.941 (3)	C18—H18D	0.9900
Mo3—O4^i^	2.451 (3)	C18—C19	1.463 (14)
Mo3—O8	2.122 (3)	C18—C17X	1.386 (18)
Mo3—O9	1.690 (3)	C18—C17	1.386 (16)
Mo3—O10	1.767 (3)	C19—H19A	0.9900
Mo3—O11	1.901 (3)	C19—H19B	0.9900
Mo4—O2^i^	2.070 (3)	C19—C20	1.529 (12)
Mo4—O8	1.994 (3)	C20—H20A	0.9900
Mo4—O11	2.284 (3)	C20—H20B	0.9900
Mo4—O12	1.706 (4)	C21—H21A	0.9900
Mo4—O13	1.734 (4)	C21—H21B	0.9900
Mo4—O14	1.987 (4)	C21—C22	1.471 (14)
Mo5—O14	1.804 (4)	C22—H22A	0.9900
Mo5—O15	1.767 (4)	C22—H22B	0.9900
Mo5—O16	1.738 (4)	C22—C23	1.476 (16)
Mo5—O17	1.741 (4)	C23—H23A	0.9900
N1—H1A	0.9100	C23—H23B	0.9900
N1—H1B	0.9100	C23—C24	1.56 (2)
N1—C4	1.479 (9)	C24—H24A	0.9800
N1—C5	1.480 (9)	C24—H24B	0.9800
C1—H1C	0.9800	C24—H24C	0.9800
C1—H1D	0.9800	C17X—H17A	0.9800
C1—H1E	0.9800	C17X—H17B	0.9800
C1—C2	1.520 (12)	C17X—H17C	0.9800
C2—H2A	0.9900	C17—H17D	0.9800
C2—H2B	0.9900	C17—H17E	0.9800
C2—C3	1.480 (13)	C17—H17F	0.9800
C3—H3C	0.9900	N4—H4A	0.9100
C3—H3D	0.9900	N4—H4B	0.9100
C3—C4	1.523 (10)	N4—C28	1.471 (11)
C4—H4C	0.9900	N4—C29	1.500 (12)
C4—H4D	0.9900	C25—H25A	0.9800
C5—H5A	0.9900	C25—H25B	0.9800
C5—H5B	0.9900	C25—H25C	0.9800
C5—C6	1.540 (10)	C25—C26	1.518 (13)
C6—H6A	0.9900	C26—H26A	0.9900
C6—H6B	0.9900	C26—H26B	0.9900
C6—C7	1.485 (13)	C26—C27	1.488 (12)
C7—H7A	0.9900	C27—H27A	0.9900
C7—H7B	0.9900	C27—H27B	0.9900
C7—C8	1.547 (14)	C27—C28	1.543 (10)
C8—H8A	0.9800	C28—H28A	0.9900
C8—H8B	0.9800	C28—H28B	0.9900
C8—H8C	0.9800	C29—H29C	0.9900
N2—H2C	0.9100	C29—H29D	0.9900
N2—H2D	0.9100	C29—H29A	0.9900
N2—C12	1.496 (8)	C29—H29B	0.9900
N2—C13	1.461 (8)	C29—C30	1.497 (11)
C9—H9A	0.9800	C29—C30X	1.502 (12)
C9—H9B	0.9800	C30—H30A	0.9900
C9—H9C	0.9800	C30—H30B	0.9900
C9—C10	1.460 (16)	C30—C31	1.486 (12)
C10—H10A	0.9900	C31—H31A	0.9900
C10—H10B	0.9900	C31—H31B	0.9900
C10—C11	1.541 (10)	C31—C32	1.500 (12)
C11—H11A	0.9900	C32—H32A	0.9800
C11—H11B	0.9900	C32—H32B	0.9800
C11—C12	1.476 (10)	C32—H32C	0.9800
C12—H12A	0.9900	C30X—H30C	0.9900
C12—H12B	0.9900	C30X—H30D	0.9900
C13—H13A	0.9900	C30X—C31X	1.502 (13)
C13—H13B	0.9900	C31X—H31C	0.9900
C13—C14	1.477 (11)	C31X—H31D	0.9900
C14—H14A	0.9900	C31X—C32X	1.502 (12)
C14—H14B	0.9900	C32X—H32D	0.9800
C14—C15	1.526 (11)	C32X—H32E	0.9800
C15—H15A	0.9900	C32X—H32F	0.9800
			
O1—Mo1—O2	101.45 (16)	N2—C13—H13B	109.0
O1—Mo1—O4	158.94 (15)	N2—C13—C14	112.9 (6)
O1—Mo1—O5	94.45 (16)	H13A—C13—H13B	107.8
O1—Mo1—O11^i^	89.75 (14)	C14—C13—H13A	109.0
O2—Mo1—O4	85.83 (12)	C14—C13—H13B	109.0
O2—Mo1—O5	150.37 (13)	C13—C14—H14A	109.3
O2—Mo1—O11^i^	74.19 (13)	C13—C14—H14B	109.3
O3—Mo1—O1	105.88 (17)	C13—C14—C15	111.6 (7)
O3—Mo1—O2	100.13 (16)	H14A—C14—H14B	108.0
O3—Mo1—O4	92.02 (14)	C15—C14—H14A	109.3
O3—Mo1—O5	99.31 (15)	C15—C14—H14B	109.3
O3—Mo1—O11^i^	164.24 (14)	C14—C15—H15A	108.9
O4—Mo1—O11^i^	73.09 (11)	C14—C15—H15B	108.9
O5—Mo1—O4	71.33 (12)	H15A—C15—H15B	107.7
O5—Mo1—O11^i^	81.10 (12)	C16—C15—C14	113.3 (10)
O4—Mo2—O10^i^	71.49 (11)	C16—C15—H15A	108.9
O5—Mo2—O4	74.75 (12)	C16—C15—H15B	108.9
O5—Mo2—O8	144.85 (13)	C15—C16—H16A	109.5
O5—Mo2—O10^i^	81.80 (12)	C15—C16—H16B	109.5
O6—Mo2—O4	152.33 (15)	C15—C16—H16C	109.5
O6—Mo2—O5	106.39 (17)	H16A—C16—H16B	109.5
O6—Mo2—O7	104.51 (17)	H16A—C16—H16C	109.5
O6—Mo2—O8	100.31 (15)	H16B—C16—H16C	109.5
O6—Mo2—O10^i^	81.24 (15)	H3A—N3—H3B	107.4
O7—Mo2—O4	102.40 (14)	C20—N3—H3A	108.3
O7—Mo2—O5	100.16 (15)	C20—N3—H3B	108.3
O7—Mo2—O8	94.73 (15)	C20—N3—C21	116.0 (6)
O7—Mo2—O10^i^	172.96 (14)	C21—N3—H3A	108.3
O8—Mo2—O4	71.01 (12)	C21—N3—H3B	108.3
O8—Mo2—O10^i^	80.09 (12)	H18A—C18—H18B	107.8
O4—Mo3—O4^i^	75.88 (12)	H18C—C18—H18D	107.6
O4—Mo3—O8	74.10 (12)	C19—C18—H18A	109.0
O8—Mo3—O4^i^	81.68 (11)	C19—C18—H18B	109.0
O9—Mo3—O4^i^	179.57 (15)	C19—C18—H18C	108.6
O9—Mo3—O4	104.54 (15)	C19—C18—H18D	108.6
O9—Mo3—O8	98.32 (15)	C17X—C18—H18A	109.0
O9—Mo3—O10	103.39 (16)	C17X—C18—H18B	109.0
O9—Mo3—O11	103.82 (15)	C17X—C18—C19	113.1 (19)
O10—Mo3—O4	97.80 (14)	C17—C18—H18C	108.6
O10—Mo3—O4^i^	76.62 (12)	C17—C18—H18D	108.6
O10—Mo3—O8	158.14 (14)	C17—C18—C19	114.5 (12)
O10—Mo3—O11	99.60 (14)	C18—C19—H19A	108.6
O11—Mo3—O4^i^	75.77 (12)	C18—C19—H19B	108.6
O11—Mo3—O4	142.06 (14)	C18—C19—C20	114.7 (9)
O11—Mo3—O8	77.31 (13)	H19A—C19—H19B	107.6
O2^i^—Mo4—O11	71.28 (12)	C20—C19—H19A	108.6
O8—Mo4—O2^i^	85.53 (13)	C20—C19—H19B	108.6
O8—Mo4—O11	71.79 (12)	N3—C20—C19	112.1 (6)
O12—Mo4—O2^i^	99.29 (17)	N3—C20—H20A	109.2
O12—Mo4—O8	94.19 (15)	N3—C20—H20B	109.2
O12—Mo4—O11	163.32 (15)	C19—C20—H20A	109.2
O12—Mo4—O13	105.07 (19)	C19—C20—H20B	109.2
O12—Mo4—O14	98.93 (17)	H20A—C20—H20B	107.9
O13—Mo4—O2^i^	155.20 (16)	N3—C21—H21A	109.5
O13—Mo4—O8	97.15 (15)	N3—C21—H21B	109.5
O13—Mo4—O11	86.08 (15)	H21A—C21—H21B	108.1
O13—Mo4—O14	90.34 (18)	C22—C21—N3	110.5 (8)
O14—Mo4—O2^i^	81.24 (15)	C22—C21—H21A	109.5
O14—Mo4—O8	162.67 (14)	C22—C21—H21B	109.5
O14—Mo4—O11	93.27 (14)	C21—C22—H22A	108.5
O15—Mo5—O14	112.07 (19)	C21—C22—H22B	108.5
O16—Mo5—O14	111.0 (2)	C21—C22—C23	115.3 (11)
O16—Mo5—O15	110.43 (19)	H22A—C22—H22B	107.5
O16—Mo5—O17	108.5 (2)	C23—C22—H22A	108.5
O17—Mo5—O14	107.55 (18)	C23—C22—H22B	108.5
O17—Mo5—O15	107.13 (18)	C22—C23—H23A	109.1
Mo1—O2—Mo4^i^	116.86 (17)	C22—C23—H23B	109.1
Mo1—O4—Mo3^i^	96.86 (11)	C22—C23—C24	112.3 (12)
Mo2—O4—Mo1	93.97 (11)	H23A—C23—H23B	107.9
Mo2—O4—Mo3^i^	96.67 (11)	C24—C23—H23A	109.1
Mo3—O4—Mo1	149.92 (16)	C24—C23—H23B	109.1
Mo3—O4—Mo2	104.56 (12)	C23—C24—H24A	109.5
Mo3—O4—Mo3^i^	104.12 (12)	C23—C24—H24B	109.5
Mo2—O5—Mo1	115.25 (16)	C23—C24—H24C	109.5
Mo2—O8—Mo3	105.76 (13)	H24A—C24—H24B	109.5
Mo4—O8—Mo2	148.17 (17)	H24A—C24—H24C	109.5
Mo4—O8—Mo3	105.24 (14)	H24B—C24—H24C	109.5
Mo3—O10—Mo2^i^	114.80 (15)	C18—C17X—H17A	109.5
Mo3—O11—Mo1^i^	114.04 (14)	C18—C17X—H17B	109.5
Mo3—O11—Mo4	102.46 (13)	C18—C17X—H17C	109.5
Mo4—O11—Mo1^i^	95.11 (12)	H17A—C17X—H17B	109.5
Mo5—O14—Mo4	165.0 (3)	H17A—C17X—H17C	109.5
H1A—N1—H1B	107.4	H17B—C17X—H17C	109.5
C4—N1—H1A	108.2	C18—C17—H17D	109.5
C4—N1—H1B	108.2	C18—C17—H17E	109.5
C4—N1—C5	116.2 (5)	C18—C17—H17F	109.5
C5—N1—H1A	108.2	H17D—C17—H17E	109.5
C5—N1—H1B	108.2	H17D—C17—H17F	109.5
H1C—C1—H1D	109.5	H17E—C17—H17F	109.5
H1C—C1—H1E	109.5	H4A—N4—H4B	107.2
H1D—C1—H1E	109.5	C28—N4—H4A	107.9
C2—C1—H1C	109.5	C28—N4—H4B	107.9
C2—C1—H1D	109.5	C28—N4—C29	117.8 (6)
C2—C1—H1E	109.5	C29—N4—H4A	107.9
C1—C2—H2A	109.1	C29—N4—H4B	107.9
C1—C2—H2B	109.1	H25A—C25—H25B	109.5
H2A—C2—H2B	107.8	H25A—C25—H25C	109.5
C3—C2—C1	112.5 (9)	H25B—C25—H25C	109.5
C3—C2—H2A	109.1	C26—C25—H25A	109.5
C3—C2—H2B	109.1	C26—C25—H25B	109.5
C2—C3—H3C	109.4	C26—C25—H25C	109.5
C2—C3—H3D	109.4	C25—C26—H26A	108.8
C2—C3—C4	111.0 (7)	C25—C26—H26B	108.8
H3C—C3—H3D	108.0	H26A—C26—H26B	107.7
C4—C3—H3C	109.4	C27—C26—C25	114.0 (8)
C4—C3—H3D	109.4	C27—C26—H26A	108.8
N1—C4—C3	112.6 (6)	C27—C26—H26B	108.8
N1—C4—H4C	109.1	C26—C27—H27A	108.9
N1—C4—H4D	109.1	C26—C27—H27B	108.9
C3—C4—H4C	109.1	C26—C27—C28	113.4 (7)
C3—C4—H4D	109.1	H27A—C27—H27B	107.7
H4C—C4—H4D	107.8	C28—C27—H27A	108.9
N1—C5—H5A	109.6	C28—C27—H27B	108.9
N1—C5—H5B	109.6	N4—C28—C27	112.8 (6)
N1—C5—C6	110.1 (6)	N4—C28—H28A	109.0
H5A—C5—H5B	108.1	N4—C28—H28B	109.0
C6—C5—H5A	109.6	C27—C28—H28A	109.0
C6—C5—H5B	109.6	C27—C28—H28B	109.0
C5—C6—H6A	109.2	H28A—C28—H28B	107.8
C5—C6—H6B	109.2	N4—C29—H29C	111.3
H6A—C6—H6B	107.9	N4—C29—H29D	111.3
C7—C6—C5	112.1 (8)	N4—C29—H29A	106.3
C7—C6—H6A	109.2	N4—C29—H29B	106.3
C7—C6—H6B	109.2	N4—C29—C30X	102.4 (16)
C6—C7—H7A	109.7	H29C—C29—H29D	109.2
C6—C7—H7B	109.7	H29A—C29—H29B	106.4
C6—C7—C8	110.0 (11)	C30—C29—N4	124.2 (13)
H7A—C7—H7B	108.2	C30—C29—H29A	106.3
C8—C7—H7A	109.7	C30—C29—H29B	106.3
C8—C7—H7B	109.7	C30X—C29—H29C	111.3
C7—C8—H8A	109.5	C30X—C29—H29D	111.3
C7—C8—H8B	109.5	C29—C30—H30A	109.3
C7—C8—H8C	109.5	C29—C30—H30B	109.3
H8A—C8—H8B	109.5	H30A—C30—H30B	108.0
H8A—C8—H8C	109.5	C31—C30—C29	111.5 (13)
H8B—C8—H8C	109.5	C31—C30—H30A	109.3
H2C—N2—H2D	107.6	C31—C30—H30B	109.3
C12—N2—H2C	108.6	C30—C31—H31A	108.5
C12—N2—H2D	108.6	C30—C31—H31B	108.5
C13—N2—H2C	108.6	C30—C31—C32	114.9 (13)
C13—N2—H2D	108.6	H31A—C31—H31B	107.5
C13—N2—C12	114.6 (5)	C32—C31—H31A	108.5
H9A—C9—H9B	109.5	C32—C31—H31B	108.5
H9A—C9—H9C	109.5	C31—C32—H32A	109.5
H9B—C9—H9C	109.5	C31—C32—H32B	109.5
C10—C9—H9A	109.5	C31—C32—H32C	109.5
C10—C9—H9B	109.5	H32A—C32—H32B	109.5
C10—C9—H9C	109.5	H32A—C32—H32C	109.5
C9—C10—H10A	109.0	H32B—C32—H32C	109.5
C9—C10—H10B	109.0	C29—C30X—H30C	109.6
C9—C10—C11	112.7 (8)	C29—C30X—H30D	109.6
H10A—C10—H10B	107.8	C29—C30X—C31X	110 (2)
C11—C10—H10A	109.0	H30C—C30X—H30D	108.1
C11—C10—H10B	109.0	C31X—C30X—H30C	109.6
C10—C11—H11A	108.8	C31X—C30X—H30D	109.6
C10—C11—H11B	108.8	C30X—C31X—H31C	109.1
H11A—C11—H11B	107.7	C30X—C31X—H31D	109.1
C12—C11—C10	113.8 (6)	C30X—C31X—C32X	112.5 (14)
C12—C11—H11A	108.8	H31C—C31X—H31D	107.8
C12—C11—H11B	108.8	C32X—C31X—H31C	109.1
N2—C12—H12A	109.1	C32X—C31X—H31D	109.1
N2—C12—H12B	109.1	C31X—C32X—H32D	109.5
C11—C12—N2	112.4 (5)	C31X—C32X—H32E	109.5
C11—C12—H12A	109.1	C31X—C32X—H32F	109.5
C11—C12—H12B	109.1	H32D—C32X—H32E	109.5
H12A—C12—H12B	107.9	H32D—C32X—H32F	109.5
N2—C13—H13A	109.0	H32E—C32X—H32F	109.5
			
O1—Mo1—O2—Mo4^i^	72.5 (2)	C5—C6—C7—C8	176.2 (8)
O3—Mo1—O2—Mo4^i^	−178.82 (17)	N2—C13—C14—C15	−177.5 (6)
O4—Mo1—O2—Mo4^i^	−87.51 (17)	C9—C10—C11—C12	−172.9 (9)
O4—Mo2—O5—Mo1	18.83 (15)	C10—C11—C12—N2	−177.6 (6)
O4—Mo3—O10—Mo2^i^	78.67 (16)	C12—N2—C13—C14	−177.5 (5)
O4^i^—Mo3—O10—Mo2^i^	5.27 (13)	C13—N2—C12—C11	−171.1 (5)
O5—Mo1—O2—Mo4^i^	−48.5 (4)	C13—C14—C15—C16	−177.6 (8)
O6—Mo2—O5—Mo1	170.08 (17)	N3—C21—C22—C23	−179.5 (12)
O7—Mo2—O5—Mo1	−81.38 (19)	C18—C19—C20—N3	168.1 (8)
O8—Mo2—O5—Mo1	32.2 (3)	C20—N3—C21—C22	170.1 (8)
O8—Mo3—O10—Mo2^i^	12.3 (4)	C21—N3—C20—C19	60.7 (9)
O9—Mo3—O10—Mo2^i^	−174.29 (16)	C21—C22—C23—C24	180.0 (14)
O10^i^—Mo2—O5—Mo1	91.78 (17)	C17X—C18—C19—C20	−95.2 (19)
O11^i^—Mo1—O2—Mo4^i^	−13.91 (15)	C17—C18—C19—C20	−176.1 (12)
O11—Mo3—O10—Mo2^i^	−67.48 (17)	N4—C29—C30—C31	64 (2)
O15—Mo5—O14—Mo4	130.5 (8)	N4—C29—C30X—C31X	168 (2)
O16—Mo5—O14—Mo4	6.5 (9)	C25—C26—C27—C28	−175.7 (8)
O17—Mo5—O14—Mo4	−112.0 (9)	C26—C27—C28—N4	178.8 (8)
N1—C5—C6—C7	−170.0 (7)	C28—N4—C29—C30	52.8 (12)
C1—C2—C3—C4	178.8 (7)	C28—N4—C29—C30X	60.7 (19)
C2—C3—C4—N1	−172.8 (6)	C29—N4—C28—C27	57.9 (9)
C4—N1—C5—C6	−77.2 (8)	C29—C30—C31—C32	63 (3)
C5—N1—C4—C3	−78.5 (7)	C29—C30X—C31X—C32X	−70 (4)

Symmetry code: (i) −*x*+1, −*y*+1, −*z*+1.

### Hydrogen-bond geometry (Å, º)

**Table:**

*D*—H···*A*	*D*—H	H···*A*	*D*···*A*	*D*—H···*A*
N1—H1*A*···O2^ii^	0.91	1.72	2.627 (6)	172
N1—H1*B*···O10^ii^	0.91	1.97	2.755 (6)	143
N2—H2*C*···O15	0.91	1.91	2.778 (6)	160
N2—H2*D*···O7^iii^	0.91	1.90	2.812 (5)	176
N3—H3*A*···O15^iv^	0.91	1.87	2.770 (6)	172
N3—H3*B*···O17	0.91	1.88	2.786 (6)	176
N4—H4*A*···O11	0.91	1.80	2.695 (6)	166
N4—H4*B*···O16	0.91	1.86	2.762 (7)	172

Symmetry codes: (ii) −*x*+2, −*y*+1, −*z*+1; (iii) −*x*+1, *y*+1/2, −*z*+3/2; (iv) −*x*+1, −*y*+1, −*z*+2.
